# Flavonoid Glycosides with a Triazole Moiety for Marine Antifouling Applications: Synthesis and Biological Activity Evaluation

**DOI:** 10.3390/md19010005

**Published:** 2020-12-24

**Authors:** Daniela Pereira, Catarina Gonçalves, Beatriz T. Martins, Andreia Palmeira, Vitor Vasconcelos, Madalena Pinto, Joana R. Almeida, Marta Correia-da-Silva, Honorina Cidade

**Affiliations:** 1 Laboratório de Química Orgânica e Farmacêutica, Departamento de Ciências Químicas, Faculdade de Farmácia, Universidade do Porto, R. Jorge de Viterbo Ferreira 228, 4050-313 Porto, Portugal; dmpereira@ff.up.pt (D.P.); beatriz.martins@itqb.unl.pt (B.T.M.); apalmeira@ff.up.pt (A.P.); madalena@ff.up.pt (M.P.); hcidade@ff.up.pt (H.C.); 2 CIIMAR—Centro Interdisciplinar de Investigação Marinha e Ambiental, Avenida General Norton de Matos, S/N, 4450-208 Matosinhos, Portugal; catarina.goncalves@ciimar.up.pt (C.G.); vmvascon@fc.up.pt (V.V.); 3 Departamento de Biologia, Faculdade de Ciências, Universidade do Porto, Rua do Campo Alegre S/N, 4169-007 Porto, Portugal

**Keywords:** flavonoids, synthesis, click chemistry, biofouling, antifouling, eco-friendly alternatives

## Abstract

Over the last decades, antifouling coatings containing biocidal compounds as active ingredients were used to prevent biofouling, and eco-friendly alternatives are needed. Previous research from our group showed that polymethoxylated chalcones and glycosylated flavones obtained by synthesis displayed antifouling activity with low toxicity. In this work, ten new polymethoxylated flavones and chalcones were synthesized for the first time, including eight with a triazole moiety. Eight known flavones and chalcones were also synthesized and tested in order to construct a quantitative structure-activity relationship (QSAR) model for these compounds. Three different antifouling profiles were found: three compounds (**1b**, **11a** and **11b**) exhibited anti-settlement activity against a macrofouling species (*Mytilus galloprovincialis*), two compounds (**6a** and **6b**) exhibited inhibitory activity against the biofilm-forming marine bacteria *Roseobacter litoralis* and one compound (**7b**) exhibited activity against both mussel larvae and microalgae *Navicula* sp. Hydrogen bonding acceptor ability of the molecule was the most significant descriptor contributing positively to the mussel larvae anti-settlement activity and, in fact, the triazolyl glycosylated chalcone 7b was the most potent compound against this species. The most promising compounds were not toxic to *Artemia salina*, highlighting the importance of pursuing the development of new synthetic antifouling agents as an ecofriendly and sustainable alternative for the marine industry.

## 1. Introduction

Marine biofouling, resulting from the accumulation of marine micro and macroorganisms on submerged surfaces, has been a huge problem for maritime industries, causing several technical and economic problems, including corrosion of materials and the increase in fuel consumption. Moreover, marine biofouling is associated with environmental and health problems, due to an increase in gas emissions and the spread of invasive species [1,2].

Biocidal paints containing organotin compounds, namely tributyltin (TBT), were widely used for decades in the maritime industry to prevent biofouling. However, due to their negative effect on the environment and on live organisms, these substances were completely banned in 2008 by the international maritime organization [3]. Since then, some booster biocides, such as Irgarol 1051 or Sea-nine 211, in combination with copper, have been used; nevertheless, even these compounds have demonstrated toxicity on living organisms. Therefore, it is imperative to find new antifouling (AF) compounds with environmentally safe characteristics [4,5,6]. Several non-toxic marine natural products with AF activity have been reported; among them, some flavonoids presented potential AF activity and low toxicity, suggesting their potential as new lead compounds for the development of new AF agents [7].

Previous works from our group reported some glycosylated flavones [8] and chalcones [9] with potential AF activity. Interestingly, when comparing the anti-settlement activity against *Mytilus galloprovincialis* of previously described chalcones, it seemed that the presence of a polymethoxylated B-ring could be important for this activity [9]. Moreover, the introduction of a triazole moiety is associated with an increase in AF activity [10]. In fact, over the last decade, there has been a great interest in the synthesis of 1,2,3-triazoles due to the fact of these moieties behaved as more than passive linkers. They carried favorable physicochemical properties, showing importance to biological activity [11,12]. This approach has been used to generate a vast array of compounds with biological potential [13,14,15,16], namely with AF activity [10,17,18]. Moreover, some antimicrobial agents are based on nitrogen heterocycles, including the triazole-based biocides fluconazole and itraconazole, which suggest their potential to act as AF agents [10].

Based on this, the present work aims to synthesize new potential AF polymethoxylated chalcone and flavone derivatives with glycosyl groups incorporating a 1,2,3-triazole moiety using a click chemistry approach. The potential of synthesized compounds as benign AF agents was assessed against the adhesive larvae of the macrofouling mussel *Mytilus galloprovincialis* and the biofilm-forming marine bacteria *Cobetia marina*, *Vibrio harveyi*, *Pseudoalteromonas atlantica*, *Halomonas aquamarina* and *Roseobacter litoralis.* The most promising compounds were submitted to complementary assays to evaluate their viability as AF agents, including the evaluation of possible mechanisms of action related with adhesion and neurotransmission pathways. These compounds were also tested for anti-microalgal activity towards *Navicula* sp. and general ecotoxicity using nauplii of the marine shrimp *Artemia salina*.

## 2. Results and Discussion

### 2.1. Synthesis and Structure Elucidation

**Scheme 1:**
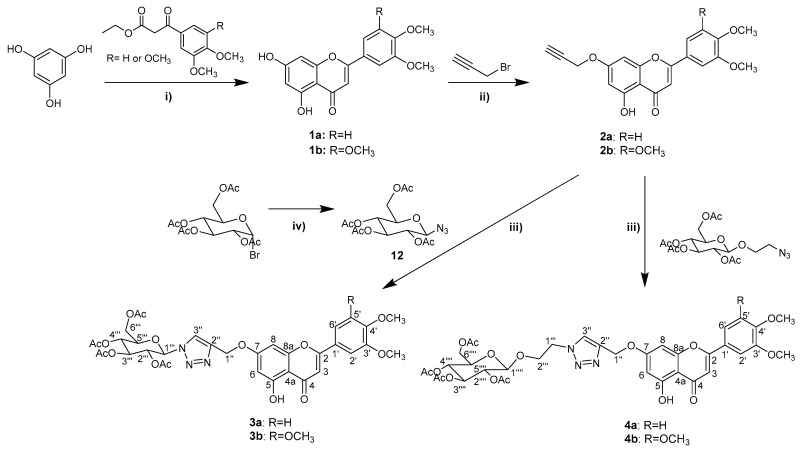
Synthesis of flavones **1a**–**1b, 2a**–**2b**, **3a**–**3b** and **4a**–**4b**. (i) 240 °C, 60–80 min, 74–77%; (ii) Cs_2_CO_3_, tetrabutylammonium bromide (TBAB), acetone, 60 °C, 6 h, 55–66%; (iii) Sodium ascorbate, CuSO_4_.5H_2_O, tetrahydrofuran (THF):water, microwave (MW), 30 min, 49–82%; (iv) NaN_3_, acetone:water, r.t., 3 h, 72%.

A series of four glycosylated flavones and four glycosylated chalcones bearing a 1,2,3-triazole moiety was synthesized. To prepare glycosylated flavones (Scheme 1), flavones **1a** and **1b**, used as building blocks, were synthesized by the Mentzer synthesis, through direct thermal cyclocondensation of phloroglucinol and β-ketoesters, with good yields, as described by Seijas et al. [19]. However, instead of a microwave (MW) irradiation, the synthesis of flavones **1a** and **1b** was performed in a muffle furnace. After, the propargylation of flavones **1a** and **1b** was achieved with propargyl bromide, giving rise to flavones **2a** and **2b** with 66% and 55% yield, respectively. Copper(I)-catalysed azide alkyne cycloaddition (CuAAC), commonly referred as click chemistry, was developed by the Sharpless and Meldal groups in 2002, and is the most useful reaction for the regioselective synthesis of 1,4-disubstituted-1,2,3-triazole ring [20,21]. This involves a reaction of a terminal alkyne and an aliphatic azide using copper (I) as a catalyst in low-time and mild conditions, with high yields and few by-products [20,21,22]. Therefore, the incorporation of the triazole linked glycosidic moiety in flavones **2a** and **2b** was accomplished by CuAAC under MW irradiation, giving rise to flavones **3a**, **3b**, **4a** and **4b** with 49–82% yield (Scheme 1).

**Scheme 2:**
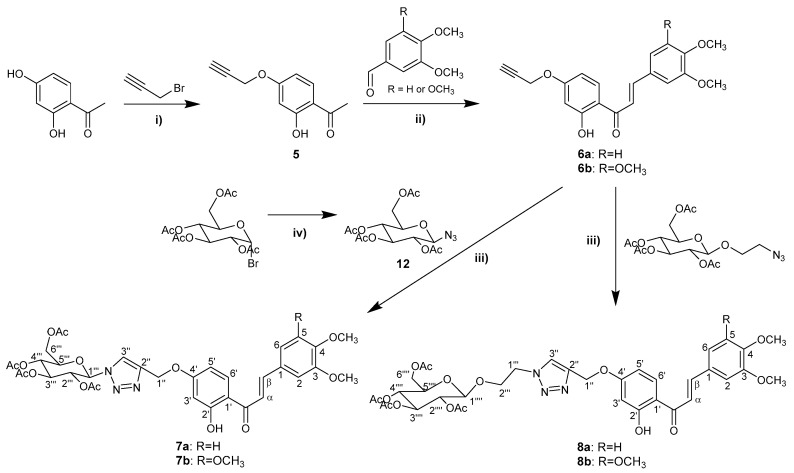
Synthesis of chalcones **6a**–**6b**, **7a**–**7b** and **8a**–**8b**. (i) K_2_CO_3_, acetone, 60 °C, 1 h, 76%; (ii) 40% NaOH, methanol, microwave (MW), 3 h, 41–43%; (iii) Sodium ascorbate, CuSO_4_.5H_2_O, tetrahydrofuran (THF):water, microwave (MW), 1 h, 45–65%; (iv) NaN_3_, acetone:water, r.t., 3 h, 72%.

The first step in the synthetic process to obtain glycosylated chalcones (Scheme 2) was the propargylation of 2,4-dihydroxyacetophenone with propargyl bromide. As for the synthesis of flavones **2a**–**2b**, firstly this reaction was accomplished with propargyl bromide, in the presence of anhydrous Cs_2_CO_3_ and tetrabutylammonium bromide (TBAB). Nevertheless, in addition to the desired 4-*O*-monosubstituted acetophenone (**5**), the 2,4-disubstituted acetophenone was obtained. Therefore, this reaction was performed in the presence of anhydrous K_2_CO_3_, as described by Zhao et al. [23], with slight modifications, and the 4-*O*-monosubstituted acetophenone (**5**) was successfully obtained as expected, with a 76% yield. Afterwards, the base-catalysed aldol reaction of this propargylated acetophenone with benzaldehydes afforded chalcones **6a** and **6b** with moderate yields, which were subsequently submitted to MW assisted CuAAC with azide sugar derivatives, affording triazole linked glycosylated chalcones **7a**, **7b**, **8a** and **8b** with 45–65% yield.

**Scheme 3:**
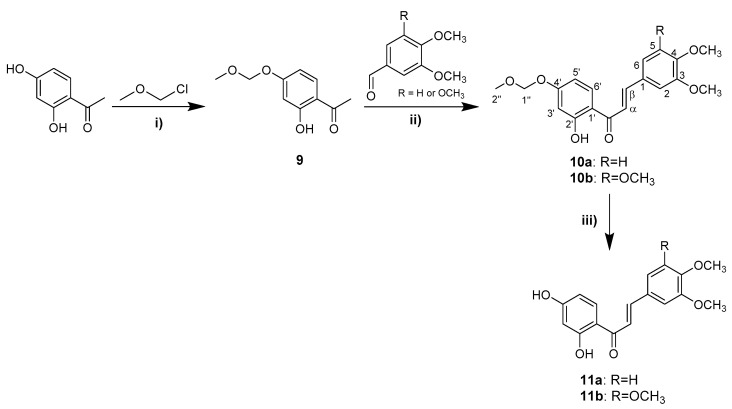
Synthesis of chalcones **10a**–**10b** and **11a**–**11b**. (i) K_2_CO_3_, acetone, 60 °C, 1 h, 84%; (ii) 40% NaOH, methanol, microwave (MW), 4 h, 33–47%; (iii) p-Toluenesulfonic acid (PTSA), methanol, 50 °C, 5 h, 24–31%.

In order to perform structure–activity relationship studies, structure related non-glycosylated chalcones were also synthesized (Scheme 3). Firstly 2,4-dihydroxyacetophenone was protected with methoxymethyl chloride affording **9** with 84% yield. Chalcones **10a** and **10b** were prepared by base-catalyzed aldol reaction of **9** and 3,4-dimethoxy- and 3,4,5-trimethoxybenzaldehyde with 33% and 47% yield, respectively, as described before [24,25], with slight modifications. Chalcones **11a** and **11b** were obtained with moderate yields by deprotection of methoxymethyl group at C-4′ of intermediate chalcones **10a** and **10b**, as described by Loureiro et al. [26].

The 2,3,4,6-tetra-*O*-acetyl-β-D-glucopyranosyl azide (**12**), used as a building block for the synthesis of glycosylated derivatives **3a**, **3b**, **7a** and **7b**, was synthesized from 2,3,4,6-tetra-*O*-acetyl-α-D-glucopyranosyl bromide and sodium azide, as described by Adesoye et al. [27], with 72% yield.

The newly synthesized compounds, **2a**, **2b**, **3a**, **3b**, **4a**, **4b**, **7a**, **7b**, **8a** and **8b** were characterized by high resolution mass spectrometry (HRMS) and nuclear magnetic resonance (NMR). The coupling constants of the vinylic system (J_Hα-Hβ_ = 15.5–15.3 Hz) confirm the (*E*)-configuration for all synthesized chalcones. The NMR spectra of the newly synthesized compounds **3a**, **3b**, **4a**, **4b**, **7a**, **7b**, **8a** and **8b** showed characteristic signals for the flavone scaffold and chalcone precursors. Additionally, signals of a triazole ring (δH-3″ 8.61–7.73 s, δC2″ 144.1–141.6 and δC3″ 125.2–121.5) and a glycosyl moiety were observed. The position of the triazole ring on these compounds was evidenced by the correlation found in the heteronuclear multiple bond correlation (HMBC) spectra between the proton signals of H-1″ and the carbon signals of C-2″ and C-3″.

### 2.2. Mussel (Mytilus galloprovincialis) Larvae Anti-Settlement Activity

Mussels are one of the main macrofouling organisms present on ships and submerged maritime structures worldwide; thus, they are a target species used in settlement inhibition bioassays [28,29]. Due to the presence of a muscular sensory foot, mussel plantigrade larvae are highly specialized in adhesion to the submerged surfaces and the fixation is made through the production of byssal threads [30], which constitutes the endpoint of this bioassay. Therefore, for the evaluation of the AF activity of the compounds towards macrofouling species, the ability of the synthetized flavonoids to inhibit the settlement of *Mytilus galloprovincialis* larvae at 50 μM was assessed. In this screening bioassay, in addition to glycosylated flavones **3a**, **3b**, **4a** and **4b** and chalcones **7a**, **7b**, **8a** and **8b**, non-glycosylated flavones **1a**–**b** and **2a**–**b** and chalcones **6a**–**b**, **10a**–**b** and **11a**–**b** were tested in order to perform SAR studies. Results showed that among 18 tested flavonoids (10 chalcones and 8 flavones), seven chalcones (**6a**, **6b**, **7b**, **8a**, **8b**, **11a** and **11b**) and only three flavones (**1b**, **4a** and **4b**) presented a percentage of settlement ≤ 40%, suggesting that chalcone scaffold seems to be more promising for anti-settlement activity. These 10 compounds were further selected for dose–response studies in order to determine LC_50_/EC_50_ values.

Among these, three chalcones (**7b**, **11a**, **11b**) and one flavone (**1b**) revealed effective anti-settlement activity (EC_50_ < 25 µg/mL), with triazolyl glycosylated chalcone **7b** being the most potent (EC_50_ = 3.28 µM; 2.43 μg·mL^−1^), showing the highest therapeutic ratio (> 60.98) (Table 1).

### 2.3. Quantitative Structure—Activity Relationship

Quantitative structure–activity relationship (QSAR) studies have been used for several years to point out small molecules’ properties that are relevant for activity, and to forecast the activity of new compounds [32]. Therefore, a QSAR model was built to highlight the descriptors that are being relevant for anti-settlement activity against *M. galloprovincialis* plantigrades of the tested flavonoids. In this work, a 2D-QSAR model was elaborated using the Comprehensive Descriptors for Structural and Statistical Analysis (CODESSA 2.7.2) software package, which calculates approximately 500 descriptors. The heuristic method performs a pre-selection of descriptors by eliminating descriptors that are not available for each structure, that have a small variation in magnitude, that are correlated pairwise, and that have no statistical significance. The heuristic method is a very useful method for searching the best set of descriptors, without restrictions on the data set size [33].

**Figure 1:**
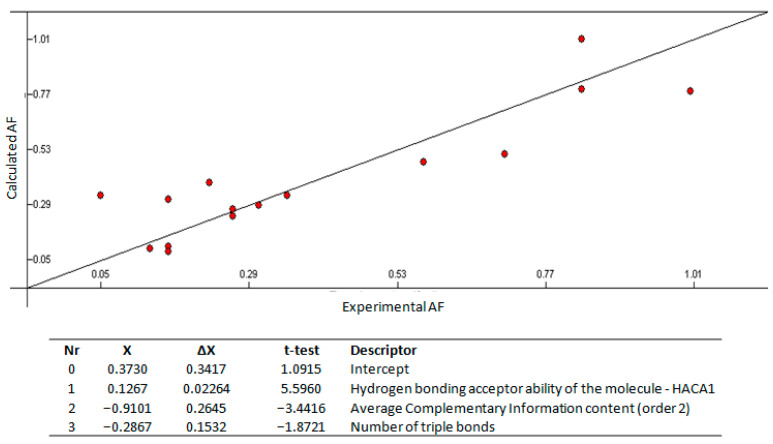
Quantitative structure-activity relationship (QSAR) model obtained with the heuristic method for 15 molecules with the CODESSA software (R^2^ = 0.7945, F = 14.18, S^2^ = 0.0243). X, ΔX and *t*-test are the regression coefficient of the linear model, standard errors of the regression coefficient, and the t significance coefficient of the determination, respectively. AF = antifouling activity.

The correlation coefficient (R^2^), squared standard error (S^2^), and Fisher’s value (F) were used to evaluate the validity of regression equation [34]. As the rules of QSAR establish that there must be one descriptor for each five molecules used to build the model [34], three descriptors were used to build the QSAR equation. The multilinear regression analysis using Heuristic method for 15 compounds in the three-descriptor model is shown in Figure 1. The compounds are uniformly distributed around the regression line (Figure 1), which suggests that the obtained model has satisfactory predictive ability.

The best QSAR equation had a R^2^ of 0.7945, Fisher value of 14.18, and S^2^ of 0.0243, which reveals that the proposed model has statistical validity [35]. The R^2^ is higher than 0.6, which is an indicator of a good fit to the regression line [36], representing close to 80% of the total variance in AF activity shown by the test compounds. The QSAR model is significant at a 95% level, as shown by the Fisher F-test (F = 14.18), which is higher than the tabulated value (3.59), as desired for a statistically significant model [35]. The squared standard deviation S^2^ is small and close to zero (s^2^ = 0.0243), proving that the model is significant and has low variation about the regression line [37]. The reliability of the resulting QSAR model was explored using two different types of validation criteria: external validation by using a test set and internal validation by leave-one-out (LOO) cross-validation [38]. The model was able to predict the activity of an external test set with an average difference of 0.19 from the experimental value [39]. Moreover, the cross-validated R^2^ (Q^2^ = 0.5953) from the LOO internal validation process is higher than 0.5 and smaller than the overall R^2^, as expected, and the difference between R^2^ and Q^2^ is lower than 0.3, which indicates that the model does not suffer from overfitting [40].

By interpreting the molecular descriptors in the regression model (Figure 1), it is possible to have some insight into structural characteristics that are likely to be responsible for AF activity of the studied compounds. There are three descriptors included in the regression model, which proved to be important features and provide statistically significant contributions to the QSAR equation.

As indicated by the higher t-test value, hydrogen bonding acceptor ability of the molecule (HACA1) is a charged partial surface area (CPSA) descriptor that appeared as the most significant descriptor for the obtained QSAR model, contributing positively to the AF activity [41]. HACA1 is determined by the equation:

(1) $$\mathrm{HACA}1=\;\underset{A}{\sum }S_{A\;}\;A\in X_{H-\mathrm{acceptor}}$$

where *S_A_* stands for solvent-accessible surface area of H-bonding acceptor atoms, selected by threshold charge. This descriptor proves the importance of the hydrogen bonding acceptor properties for the activity of the test compounds [42].

The topological descriptor average complementary information content of order 2 (CIC2) descriptor is predicted as being negatively implied in the AF activity of the test compounds [41]. The CIC2 descriptor represents the difference between the maximum possible complexity of a molecule and its real topological information. It belongs to the multi-graph information content indices and it describes neighborhood symmetry of second order [43]. The constitutional descriptor number of triple bonds is also responsible for a decrease in activity.

The molecular descriptors used in the QSAR model demonstrate that the mechanism underlying the AF activity of flavonoids is mainly related to their HACA1, and it may be prejudiced by topological CIC2 and by the presence of triple bonds. Interestingly, the triazolyl glycosylated chalcone **7b**, with the most promising anti-settlement activity, is one of the compounds with more hydrogen-bonding acceptors. In contrast, propargylated flavones **2a** and **2b** had a percentage of settlement higher than 40% at 50 µM, and therefore were not selected for dose–response studies and for the determination of the LC_50_/EC_50_ values. Moreover, propargylated chalcones **6a** and **6b** showed the lowest activity. Overall, the examination of the molecular descriptors reported in this work can lead to a better understanding of the relation between the structure and AF activity of flavonoids.

### 2.4. Biofilm-Forming Marine Bacteria Growth Inhibitory Activity

Although the macrofouling species represent the most problematic component of fouling in terms of biomass and negative repercussions, the first micro-colonizers are also of extreme importance, since they represent the basis of the fouling community, and ultimately, they may modulate the colonization of further species by inducing or inhibiting species adhesion via biochemical cues [44]. Thus, synthesized flavonoids were further evaluated for their ability to inhibit the growth of five marine biofilm-forming bacteria, *Vibrio harveyi, Cobetia marina, Halomonas aquamarina, Pseudoalteromonas atlantica* and *Roseobacter litoralis*.

**Figure 2:**
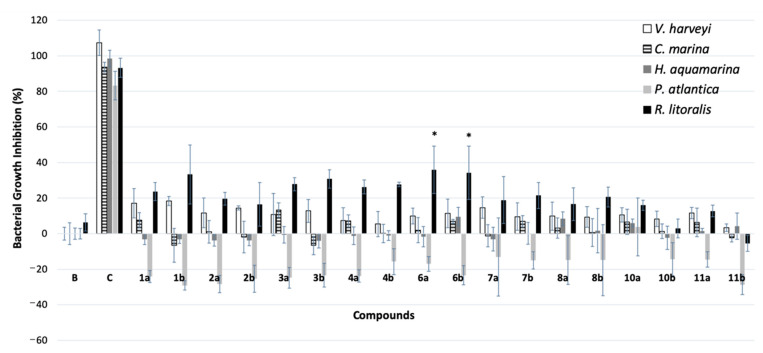
Bacterial growth inhibition screening of flavonoid derivatives (15 µM) towards five biofilm-forming marine bacteria: *Vibrio harveyi*, *Cobetia marina*, *Halomonas aquamarina*, *Pseudoalteromonas atlantica* and *Roseobacter litoralis*. B: Negative control with 1% dimethyl sulfoxide (DMSO); C: positive control with penicillin–streptomycin–neomycin-stabilized solution. * indicates significant differences at *p* < 0.05 (Dunnett test), against the negative control (B).

**Figure 3:**
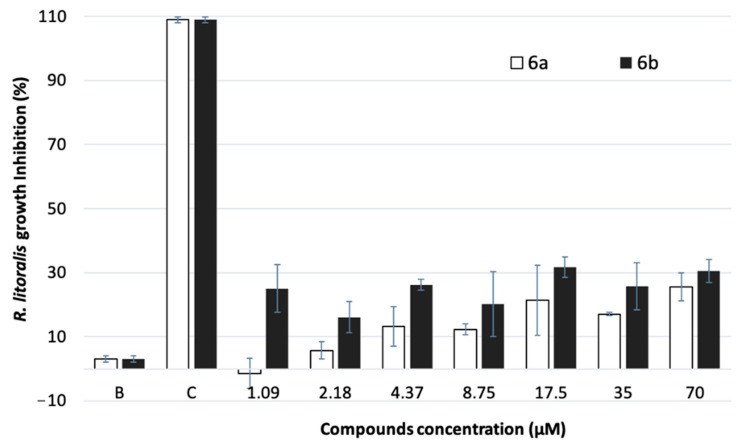
Concentration–response growth inhibition activity of compounds **6a** and **6b** towards *Roseobacter litoralis*. B: DMSO negative control; C: positive control with penicillin–streptomycin–neomycin-stabilized solution.

Results showed that only the bacterial growth of *Roseobacter litoralis* was meaningfully compromised by tested compounds, with significant inhibitory activity for propargylated chalcones **6a** and **6b** (Figure 2). These compounds were selected for concentration–response analysis (Figure 3).

Compounds **6a** and **6b** presented low anti-bacterial activity towards *R. litoralis* with EC_30_ values of 135 and 83.5 µM, respectively.

### 2.5. Biofilm—Forming Marine Diatoms Growth Inhibitory Activity

The most promising compounds regarding anti-settlement activity (**1b**, **7b**, **11a**, **11b**) were further evaluated for their ability to inhibit the growth of the biofilm-forming microalgae *Navicula* sp. This marine diatom is a major biofouling species that very effectively colonizes submerged surfaces by secreting adhesive extracellular polymer substances (EPS), and thus is a good representative of fouling microalgae.

Only triazolyl glycosylated chalcone **7b** showed significant inhibitory activity with the concentration–response analyses revealing an EC_50_ value of 41.76 µM; 30.94 µg·mL^−1^, suggesting the ability of this compound to act also as a promising AF agent against microfouling species.

### 2.6. In Vitro Acetylcholinesterase (AChE) and Tyrosinase (Tyr) Activities

**Figure 4:**
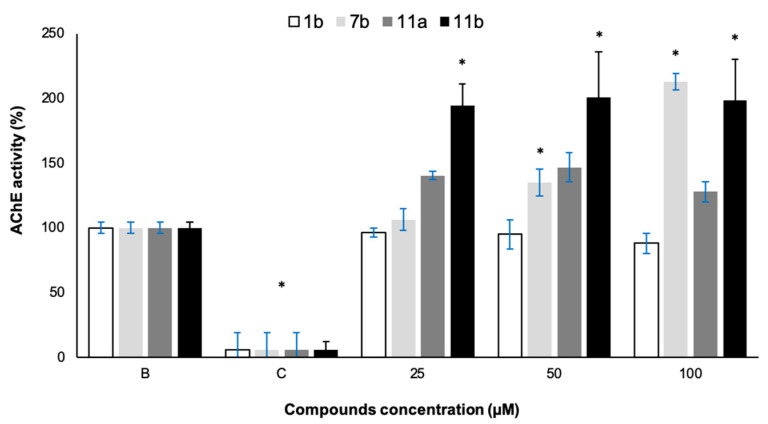
AChE activity of the most promising compounds **1b 7b**, **11a** and **11b**. B: Dimethyl sulfoxide (DMSO) (1% in water). C: Eserine (200 μM, water). * indicates significant differences at *p* < 0.05 (Dunnett test), against the negative control (B).

The identification of the mechanism of action associated with AF activity remains a challenge for the scientific community. According to Qian et al. (2013) antifoulants appear to affect settlement through distinct pathways, which can be classified roughly into several categories such as inhibitors of ion channel function, inhibitors of quorum sensing, blockers of neurotransmission or inhibitors of adhesive production or release [45]. Moreover, some specific target molecules in fouling organisms have been determined, such as AChE, which seems to be involved in cholinergic neural signaling during the settlement [46]. It is known that the commercial booster biocide Sea-Nine 211 acts by this mechanism [47,48], as well as two natural compounds isolated from marine organisms, territrem A and pulmonarin [49,50]. For this reason, the ability of the most promising compounds to modulate the activity of AChE was evaluated (**1b**, **7b**, **11a** and **11b**). AChE activity was significantly induced for chalcones **7b** and **11b** (Figure 4). Induced AChE activity has been described as an exposure effect that is in some cases associated with apoptosis [51], and thus the specific target behind these compounds’ bioactivity should be further explored in future work.

**Figure 5:**
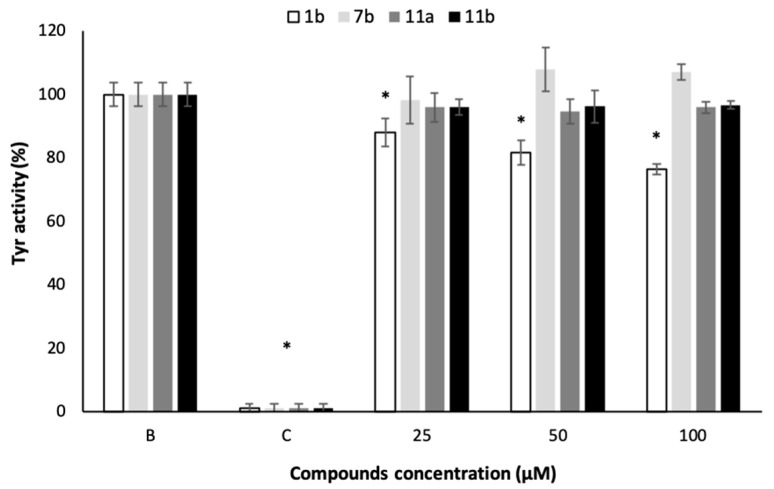
Tyr activity of the most promising compounds **1b, 7b**, **11a** and **11b**. B: Dimethyl sulfoxide (DMSO) (1% in water). C: Kojic acid (1.4 mM, water). * indicates significant differences at *p* < 0.05 (Dunnett test), against the negative control (B).

A well-known pathway in the production of biological adhesives of mussels is the 3,4-dihydroxyphenyl-L-alanine (L-DOPA) metabolism that functions in the production of DOPA-containing mussel byssal plaques by the action of Tyr that catalyses the conversion of DOPA precursor into DOPA residues [46,52]. Considering this, the most promising compounds in the inhibition of mussel adhesion were tested for their ability to inhibit Tyr (Figure 5). Results show that flavone **1b** is able to significantly decrease Tyr activity at all the concentrations tested, reaching 23.5% of inhibition at 100 µM. Therefore, the inhibition of this enzyme, with a crucial effect in the formation of mussel adhesive, could be one of the mechanisms involved in the inhibition of the mussel settlement. This also highlights a specific AF mode of action related with mussel adhesion and explains the absence of activity against bacteria and diatoms.

### 2.7. Environmental Fate Parameters: Artemia Salina Ecotoxicity Bioassay

Ecotoxicity assays carried out on non-target organisms aim to understand how tested compounds can affect sensitive non-target organisms and influence the health status of the surrounding ecosystem [53]. *Artemia salina* is a species of small crustaceans that live in salty marine environments and are used as test organisms because of their easy culture, short generation time, cosmopolitan distribution and commercial availability of their eggs in latent form [54].

**Figure 6:**
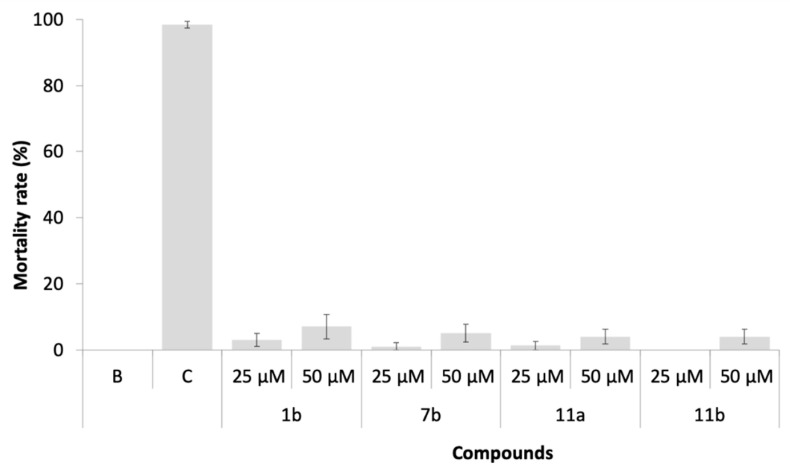
Mortality rate of *Artemia salina* nauplii after 48 h of exposure to compounds **1b**, **7b**, **11a** and **11b**, B: DMSO (1% in filtered seawater). C: K_2_Cr_2_O_7_ (13.6 μM, filtered seawater).

Ecotoxicity results showed that the most promising compounds **1b**, **7b**, **11a** and **11b** are non-toxic to *Artemia salina* (less than 10% mortality) at both concentrations tested (25 and 50 μM) (Figure 6), in contrast to the commercial AF agent ECONEA^®^ which was previously shown by our group to cause 100% lethality at the same concentrations and conditions [9]. These results suggest that any of the tested compounds could be a good alternative, being more environmentally compatible antifoulants.

## 3. Materials and Methods

### 3.1. Synthesis and Structure Elucidation of Chalcones and Flavones

MW reactions were performed using a glassware setup for atmospheric pressure reactions and a 100 mL Teflon reactor (internal reaction temperature measurements with a fiber-optic probe sensor) and were carried out using an Ethos MicroSYNTH 1600 Microwave Labstation from Milestone (Thermo Unicam, Oeiras, Portugal). The reactions were monitored by analytical thin-layer chromatography (TLC) Macherey-Nagel Silica gel 60 F254 (Macherey-Nagel, Dueren, Germany), Purifications of compounds were carried out by flash chromatography using Macherey-Nagel silica gel 60 (0.04–0.063 mm) (Macherey-Nagel, Dueren, Germany), preparative TLC using Macherey-Nagel silica gel 60 (GF254) (Macherey-Nagel, Dueren, Germany) plates and crystallization. Melting points were obtained in a Köfler microscope (Wagner and Munz, Munich, Germany) and are uncorrected. ^1^H and ^13^C NMR spectra were taken in CDCl_3_ or DMSO-d_6_ at room temperature, on Bruker Avance 300 and 500 instruments (Bruker Biosciences Corporation, Billerica, MA, USA) (300.13 MHz or 500 MHz for ^1^H and 75.47 or 120 MHz for ^13^C). Chemical shifts are expressed in δ (ppm) values relative to tetramethylsilane (TMS) as an internal reference; ^13^C NMR assignments were made by 2D (HSQC and HMBC) NMR experiments (long-range ^13^C-^1^H coupling constants were optimized to 7 Hz). HRMS mass spectra of compounds **2a**, **2b**, **3a**, **3b**, **4a**, **4b**, **7a**, **7b** and **8b** were performed on an APEXQe FT-ICR MS (Bruker Daltonics, Billerica, MA) equipped with a 7T actively shielded magnet, at C.A.C.T.I.—University of Vigo, Spain. Ions were generated using a Combi MALDI-electrospray ionization (ESI) source. HRMS mass spectrometry of compound **8a** was performed on an LTQ Orbitrap^TM^ XL hybrid mass spectrometer (Thermo Fischer Scientific, Bremen, Germany) controlled by LTQ Tune Plus 2.5.5 and Xcalibur 2.1.0. at CEMUP—University of Porto, Portugal. Phloroglucinol, ethyl 3,4-dimethoxybenzoylacetate, ethyl 3,4,5-trimethoxybenzoylacetate, 2,4-dihydroxyacetophenone and 2,3,4,6-tetra-*O*-acetyl-α-D-glucopyranosyl bromide were purchased from Sigma Aldrich (St. Louis, MO, USA). 3,4-Dimethoxybenzaldehyde and 3,4,5-trimethoxybenzaldehyde were purchased from Acros Organics (Janssen Pharmaceuticalaan, Geel, Belgium). 2-Azidoethyl-2,3,4,6-tetra-*O*-acetyl-β-D-glucopyranoside was purchased from Synthose (Concord, ON, Canada).

#### 3.1.1. Synthesis of Flavones **1a** and **1b**

A mixture of phloroglucinol (0.175 g, 1.39 mmol) and ethyl 3,4-dimethoxybenzoylacetate (0.700 g, 2.78 mmol) or ethyl 3,4,5-trimethoxybenzoylacetate (0.739 g, 2.78 mmol) was heated at 240 °C in muffle furnace (Thermo Fisher Scientific, Oeiras, Portugal) for 60–100 min. Afterwards, the crude mixture was dissolved in 10% NaOH (20 mL) and washed with diethyl ether (2 × 20 mL), and the product was precipitated by adding 37% HCl. The solid was filtered and washed with water, and the flavones **1a** and **1b** were obtained with 74% and 77% yields, respectively. The structure elucidation of compounds **1a** and **2b** was established by ^1^H and ^13^C NMR techniques and data were in accordance with previously reported results [19].

#### 3.1.2. Synthesis of 7-*O*-Propargylflavones **2a** and **2b**

To a solution of **1a** (0.200 g, 0.64 mmol) or **1b** (0.200 g, 0.58 mmol), cesium carbonate (0.207 g, 0.64 mmol or 0.189 g, 0.58 mmol), tetrabutylammonium bromide (TBAB) (0.205 g, 0.64 mmol or 0.187 g, 0.58 mmol) in anhydrous acetone (20 mL), and propargyl bromide solution, 80 wt.% in toluene (0.071 mL, 0.64 mmol or 0.065 mL, 0.58 mmol), were added. The mixture was refluxed at 60 °C during 6 h and filtered. The filtrate was evaporated under reduced pressure and purification was carried out by flash column chromatography (SiO_2_; n-hexane: ethyl acetate), 8:2) followed by crystallization in acetone.

2-(3,4-dimethoxyphenyl)-5-hydroxy-7-(prop-2-yn-1-yloxy)-4H-chromen-4-one (**2a**). Light yellow solid; Yield: 66%; m.p.: 222–224 °C (acetone); ^1^H NMR (DMSO-d_6_, 500 MHz), δ: 12.93 (1H, s, OH-5), 7.71 (1H, dd, J = 8.5 and 2.2 Hz, H-6′), 7.57 (1H, d, J = 2.2 Hz, H-2′), 7.14 (1H, d, J = 8.7 Hz, H-5′), 7.03 (1H, s, H-3), 6.87 (1H, d, J = 2.2 Hz, H-8), 6.44 (1H, d, J = 2.3 Hz, H-6), 4.94 (2H, d, J = 2.5 Hz, H-1″), 3.88 (3H, s, 3′-OCH_3_), 3.85 (3H, s, 4′-OCH_3_), 3.66 (1H, t, J = 2.4 Hz, H-3″). ^13^C NMR (DMSO-d_6_, 120 MHz) δ: 182.3 (C4), 164.0 (C2), 163.2 (C7), 161.3 (C5), 157.3 (C8a), 152.5 (C4′), 149.2 (C3′), 122.9 (C1′), 120.5 (C6′), 111.9 (C5′), 109.6 (C2′), 105.3 (C4a), 104.3 (C3), 98.8 (C6), 94.0 (C8), 79.2 (C3″), 78.6 (C2″), 56.4 (C1″), 56.1, 56.0 (3′-OCH_3_ and 4′-OCH_3_). HRMS (ESI^+^) *m*/*z* calcd for C_20_H_17_O_6_ [M + H^+^] 353.10196, found 353.10174.

5-hydroxy-7-(prop-2-yn-1-yloxy)-2-(3,4,5-trimethoxyphenyl)-4H-chromen-4-one (**2b**). Light yellow solid; Yield: 55%; m.p.: 211–213 °C (acetone); ^1^H NMR (DMSO-d_6_, 300.13 MHz), δ: 12.87 (1H, s, OH-5), 7.36 (2H, s, H-2′ and H-6′), 7.16 (1H, s, H-3), 6.91 (1H, d, J = 2.3 Hz, H-8), 6.46 (1H, d, J = 2.3 Hz, H-6), 4.96 (2H, d, J = 2.4 Hz, H-1″), 3.91 (6H, s, 3′-OCH_3_ and 5′-OCH_3_), 3.76 (3H, s, 4′- OCH_3_), 3.70 (1H, t, J = 2.4 Hz, H-3″). ^13^C NMR (DMSO-d_6_, 75.47 MHz) δ: 182.2 (C4), 163.4 (C2), 163.1 (C7), 161.2 (C5), 157.1 (C8a), 153.3 (C3′ and C5′), 140.9 (C4′), 125.8 (C1′), 105.3 (C3), 105.2 (C4a), 104.2 (C2′ and C6′), 98.7 (C6), 93.9 (C8), 79.1 (C2″), 78.4 (C3″), 60.3 (4′-OCH_3_), 56.4 (C1″, 3′-OCH_3_ and 5′-OCH_3_). HRMS (ESI^+^) *m*/*z* calcd for C_21_H_19_O_7_ [M + H^+^] 383.11253, found 383.11233.

#### 3.1.3. Synthesis of Flavone-Triazolyl-Glycosides **3a** and **3b**

To a solution of **2a** (0.100 g, 0.28 mmol) or **2b** (0.100 g, 0.27 mmol) and 2,3,4,6-tetra-*O*-acetyl-β-D-glucopyranosyl azide (0.106 g, 0.28 mmol or 0.102 g, 0.27 mmol) in THF/water solvent mixture (2:1; 30 mL), sodium ascorbate (0.225 g, 1.14 mmol or 0.216 g, 1.09 mmol) and copper(II) sulphate pentahydrate (0.142 g, 0.57 mmol or 0.136 g, 0.54 mmol) were added. The reaction vessel was sealed and the mixture was kept stirring and heated for 30 min at 70 °C under MW irradiation of 500 W. After cooling, the reaction mixture was filtered and concentrated under reduced pressure. The water suspension was extracted with ethyl acetate (2 × 20 mL), and the combined organic layers were dried over anhydrous sodium sulphate, evaporated under reduced pressure, and then purified by crystallization in acetone.

(*2R*,*3R*,*4S*,*5R*,*6R*)-2-(acetoxymethyl)-6-(4-(((2-(3,4-dimethoxyphenyl)-5-hydroxy-4-oxo-4H-chromen-7-yl)oxy)methyl)-1H-1,2,3-triazol-1-yl)tetrahydro-2H-pyran-3,4,5-triyl triacetate (**3a**). Light yellow solid; Yield: 82%; m.p.: 143–145 °C (acetone); ^1^H NMR (DMSO-d_6_, 500 MHz), δ: 12.93 (1H, s, OH-5), 8.61 (1H, s, H-3″), 7.71 (1H, dd, J = 8.5 and 2.1 Hz, H-6′), 7.60 (1H, d, J = 2.2 Hz, H-2′), 7.15 (1H, d, J = 8.7 Hz, H-5′), 7.05 (1H, s, H-3), 6.94 (1H, d, J = 2.2 Hz, H-8), 6.47 (1H, d, J = 2.2 Hz, H-6), 6.39 (1H, d, J = 9.2 Hz, H-1‴), 5.68 (1H, t, J = 9.4 Hz), 5.56 (1H, t, J = 9.5 Hz), 5.19 (1H, t, J = 9.8 Hz) (H-2‴, H-3‴, H-4‴), 5.32 (2H, s, H-1″), 4.39–4.36 (1H, m, H-5‴), 4.15–4.07 (2H, m, H-6‴), 3.86 (3H, s, 4′-OCH_3_), 3.83 (3H, s, 3′-OCH_3_), 2.03, 1.99, 1.96, 1.77 (12H, s, 2‴, 3‴, 4‴, -COCH_3_). ^13^C NMR (DMSO-d_6_, 120 MHz) δ: 182.1 (C4), 170.1, 169.6, 169.4, 168.5 (2‴, 3‴, 4‴, 6‴-COCH_3_), 163.7 (C2 and C7), 161.2 (C5), 158.0 (C8a), 152.3 (C4′), 149.1 (C3′), 142.7 (C2″), 124.0 (C3″), 122.7 (C1′), 120.2 (C6′), 111.7 (C5′), 109.5 (C2′), 105.0 (C3), 104.1 (C4a), 98.7 (C6), 93.7 (C8), 83.9 (C1‴), 73.3 (C5‴), 72.1, 70.1, 67.5 (C2‴, C3‴, C4‴), 61.8 (C6‴), 61.7 (C1″), 55.9, 55.8 (3′-OCH_3_ and 4′-OCH_3_), 20.5, 20.4, 20.3, 19.9 (2‴, 3‴, 4‴, 6‴-COCH_3_). HRMS (ESI^+^) *m*/*z* calcd for C_34_H_36_N_3_O_15_ [M + H^+^] 726.21409, found 726.21380.

(*2R*,*3R*,*4S*,*5R*,*6R*)-2-(acetoxymethyl)-6-(4-(((5-hydroxy-4-oxo-2-(3,4,5-trimethoxyphenyl)-4H-chromen-7-yl)oxy)methyl)-1H-1,2,3-triazol-1-yl)tetrahydro-2H-pyran-3,4,5-triyl triacetate (**3b**). Yellow solid; Yield: 79%; m.p.: 169–171 °C (acetone); ^1^H NMR (DMSO-d_6_, 300.13 MHz), δ: 12.85 (1H, s, OH-5), 8.61 (1H, s, H-3″), 7.38 (2H, s, H-2′ and H-6′), 7.17 (1H, s, H-3), 7.00 (1H, d, J = 2.2 Hz, H-8), 6.49 (1H, d, J = 2.2 Hz, H-6), 6.39 (1H, d, J = 9.1 Hz, H-1‴), 5.68 (1H, t, J = 9.3 Hz), 5.56 (1H, t, J = 9.4 Hz), 5.19 (1H, t, J = 9.7 Hz) (H-2‴, H-3‴, H-4‴), 5.33 (2H, s, H-1″), 4.40–4.36 (1H, m, H-5‴), 4.17–4.06 (2H, m, H-6‴), 3.91 (6H, s, 3′-OCH_3_ and 5′-OCH_3_), 3.76 (3H, s, 4′-OCH_3_), 2.03, 2.00, 1.96, 1.77 (12H, s, 2‴, 3‴, 4‴, 6‴-COCH_3_). ^13^C NMR (DMSO-d_6_, 75.47 MHz) δ: 182.2 (C4), 170.1, 169.6, 169.4, 168.5 (2‴, 3‴, 4‴, 6‴-COCH_3_), 163.8 (C7), 163.4 (C2), 161.2 (C5), 157.3 (C8a), 153.3 (C3′ and C5′), 142.7 (C2″), 140.9 (C4′), 125.8 (C1′), 123.9 (C3″), 105.1 (C3 and C4a), 104.2 (C2′ and C6′), 98.7 (C6), 93.9 (C8), 83.9 (C1‴), 73.3 (C5‴), 72.1, 70.1, 67.5 (C2‴, C3‴, C4‴), 61.8 (C1″ and C6‴), 60.3 (4′-OCH_3_), 56.3 (3′-OCH_3_ and 5′-OCH_3_), 20.5, 20.4, 20.3, 19.9 (2‴, 3‴, 4‴, 6‴-COCH_3_). HRMS (ESI^+^) *m*/*z* calcd for C_35_H_38_N_3_O_16_ [M + H^+^] 756.22466, found 756.22445.

#### 3.1.4. Synthesis of Flavone-Triazolyl-Glycosides **4a** and **4b**

To a solution of **2a** (0.090 g, 0.26 mmol) or **2b** (0.100 g, 0.26 mmol) and 2-azidoethyl-2,3,4,6-tetra-*O*-acetyl-β-D-glucopyranoside (0.109 g, 0.26 mmol) in tetrahydrofuran/water solvent mixture (2:1; 30 mL), sodium ascorbate (0.207 g, 1.05 mmol) and copper(II) sulphate pentahydrate (0.131 g, 0.52 mmol) were added. The reaction vessel was sealed and the mixture was kept stirring and heated for 30 min at 70 °C under MW irradiation of 500 W. After cooling, the reaction mixture was filtered and concentrated under reduced pressure. The water suspension was extracted with ethyl acetate (2 × 20 mL), and the combined organic layers were dried over anhydrous sodium sulphate, evaporated under reduced pressure, and then purified by crystallization in ethyl acetate/n-hexane (**4a**) or by flash column chromatography (SiO_2_; n-hexane: ethyl acetate, 3:7) (**4b**).

(*2R*,*3R*,*4S*,*5R*,*6R*)-2-(acetoxymethyl)-6-(2-(4-(((2-(3,4-dimethoxyphenyl)-5-hydroxy-4-oxo-4H-chromen-7-yl)oxy)methyl)-1H-1,2,3-triazol-1-yl)ethoxy)tetrahydro-2H-pyran-3,4,5-triyl triacetate (**4a**). Light brown solid; Yield: 60%; m.p.: 99–100 °C (n-hexane: ethyl acetate); ^1^H NMR (DMSO-d_6_, 300.13 MHz), δ: 12.94 (1H, s, OH-5), 8.15 (1H, s, H-3″), 7.72 (1H, dd, J = 8.5, 1.8 Hz, H-6′), 7.59 (1H, d, J = 1.9 Hz, H-2′), 7.14 (1H, d, J = 8.6 Hz, H-5′), 7.05 (1H, s, H-3), 6.96 (1H, d, J = 2.1 Hz, H-8), 6.47 (1H, d, J = 2.1 Hz, H-6), 5.28 (2H, s, H-1″), 5.23 (1H, t, J = 9.6 Hz), 4.90 (1H, t, J = 9.7 Hz), 4.77–4.71 (1H, m) (H-2⁗, H-3⁗, H-4⁗), 4.72 (1H, d, J = 8.1 Hz, H-1⁗), 4.60–4.57 (2H, m, H-6⁗), 4.19–4.01 (5H, m, H-1‴, H-2‴, H-5⁗), 3.89 (3H, s, 3′-OCH_3_), 3.86 (3H, s, 4′-OCH_3_), 2.02, 1.98, 1.92, 1.89 (12H, s, 2⁗, 3⁗, 4⁗, 6⁗-COCH_3_). ^13^C NMR (DMSO-d_6_, 75.47 MHz) δ: 182.1 (C4), 170.1, 169.6, 169.3, 169.0 (2⁗, 3⁗, 4⁗, 6⁗-COCH_3_), 163.9 (C7), 163.7 (C2), 161.2 (C5), 157.2 (C8a), 152.3 (C4′), 149.0 (C3′), 141.7 (C2″), 125.2 (C3″), 122.8 (C1′), 120.2 (C6′), 111.7 (C5′), 109.5 (C2′), 105.0 (C4a), 104.1 (C3), 99.2 (C1⁗), 98.6 (C6), 93.5 (C8), 71.9, 70.7, 68.1 (C2⁗, C3⁗, C4⁗), 70.6, 67.4, 61.7 (C1‴, C2‴, C5⁗), 61.9 (C1″), 55.9, 55.8 (3′-OCH_3_ and 4′-OCH_3_), 49.4 (C6⁗), 20.5, 20.4, 20.3, 20.3 (2⁗, 3⁗, 4⁗, 6⁗-COCH_3_). HRMS (ESI^+^) *m*/*z* calcd for C_36_H_40_N_3_O_16_ [M + H^+^] 770.24031, found 770. 23792.

(*2R*,*3R*,*4S*,*5R*,*6R*)-2-(acetoxymethyl)-6-(2-(4-(((5-hydroxy-4-oxo-2-(3,4,5-trimethoxyphenyl)-4H-chromen-7-yl)oxy)methyl)-1H-1,2,3-triazol-1-yl)ethoxy)tetrahydro-2H-pyran-3,4,5-triyl triacetate (**4b**). Light yellow solid; Yield: 49%; m.p.: 96–98 °C (n-hexane: ethyl acetate); ^1^H NMR (DMSO-d_6_, 300.13 MHz), δ: 12.86 (1H, s, OH-5), 8.15 (1H, s, H-3″), 7.38 (2H, s, H-2′ and H-6′), 7.17 (1H, s, H-3), 7.02 (1H, d, J = 2.2 Hz, H-8), 6.49 (1H, d, J = 2.2 Hz, H-6), 5.29 (2H, s, H-1″), 5.23 (1H, t, J = 9.5 Hz), 4.90 (1H, t, J = 9.7 Hz), 4.74 (1H, t, J = 8.8 Hz) (H-2⁗, H-3⁗, H-4⁗), 4.84 (1H, d, J = 8.0, H-1⁗), 4.60–4.56 (2H, m, H-6⁗), 4.21–4.01 (5H, m, H-5⁗, H-1‴ and H-2‴), 3.91 (6H, s, 3′-OCH_3_ and 5′-OCH_3_), 3.76 (3H, s, 4′-OCH_3_), 2.02, 1.98, 1.92, 1.89 (12H, s, 2⁗, 3⁗, 4⁗, 6⁗-COCH_3_). ^13^C NMR (DMSO-d_6_, 75.47 MHz) δ: 182.2 (C4), 170.1, 169.6, 169.3, 169.0 (2⁗, 3⁗, 4⁗, 6⁗-COCH_3_), 164.0 (C7), 163.4 (C2), 161.1 (C5), 157.3 (C8a), 153.3 (C3′ and C5′), 141.6 (C2″), 140.9 (C4′), 125.8 (C1′), 125.2 (C3″), 105.3 (C4a), 105.1 (C3), 104.2 (C2′ and C6′), 99.2 (C1⁗), 98.7 (C6), 93.7 (C8), 71.9, 70.7, 70.6, 68.1 (C2⁗, C3⁗, C4⁗ and C5⁗), 67.4, 61.7 (C1‴, C2‴), 62.0 (C1″), 60.3 (4′-OCH_3_), 56.4 (3′ and 5′-OCH_3_), 49.4 (C6⁗), 20.5, 20.4, 20.3, 20.3 (2⁗, 3⁗, 4⁗, 6⁗-COCH_3_). HRMS (ESI^+^) *m*/*z* calcd for C_37_H_42_N_3_O_17_ [M + H^+^] 800.25087, found 800.24829.

#### 3.1.5. Synthesis of Propargyloxyacetophenone **5**

To a solution of 2,4-dihydroxyacetophenone (1.00 g, 6.57 mmol) and potassium carbonate (0.91 g, 6.57 mmol) in anhydrous acetone (20 mL), propargyl bromide solution, 80 wt.% in toluene (0.73 mL, 6.57 mmol) was added. The mixture was refluxed at 60 °C during 3 h. Then, the reaction mixture was filtered, evaporated under reduced pressure and purified by flash column chromatography (SiO_2_; n-hexane: ethyl acetate), 9:1), giving rise to **5** with 76% yield. The structure elucidation of compound **5** was established by ^1^H and ^13^C NMR techniques and data were in accordance with previously reported results [23].

#### 3.1.6. Synthesis of Propargyloxychalcones **6a** and **6b**

To a solution of **5** (0.350 g, 1.84 mmol) in methanol (20 mL) was added a solution of 40% NaOH in methanol, until pH 14, under stirring. Afterwards, 3,4-dimethoxybenzaldehyde (0.612 g, 3.68 mmol) or 3,4,5-trimethoxybenzaldehyde (0.772 g, 3.68 mmol) was slowly added to the reaction mixture. The reaction was submitted to MW irradiation at 180 W at 70 °C for 4 h. After, a solution of 10% HCl was added until pH 5, and the obtained solid was filtered, washed with water, and purified by crystallization with methanol, giving rise to chalcone **6a** and **6b** with 41% and 43% yield, respectively. The structure elucidation of compounds **6a** and **6b** was established by ^1^H and ^13^C NMR techniques and data were in accordance with previously reported results [23].

#### 3.1.7. Synthesis of Chalcone-Triazolyl-Glycosides **7a** and **7b**

To a solution of **6a** (0.140 g, 0.41 mmol) or **6b** (0.200 g, 0.54 mmol) and 2,3,4,6-tetra-*O*-acetyl-β-D-glucopyranosyl azide (0.309 g, 0.82 mmol or 0.405 g, 1.09 mmol) in tetrahydrofuran (THF)/water solvent mixture (2:1; 30 mL), sodium ascorbate (0.328 g, 1.66 mmol or 0.430 g, 2.17 mmol) and copper(II) sulphate pentahydrate (0.207 g, 0.83 mmol or 0.271 g, 1.09 mmol) were added. The reaction mixture was heated for 1 h at 70 °C under MW irradiation of 250 W with agitation. After cooling, the reaction mixture was filtered and concentrated under reduced pressure. The water suspension was extracted with ethyl acetate (2 × 20 mL) and the combined organic layers were washed with water (1 × 20 mL), dried over anhydrous sodium sulphate, evaporated under reduced pressure, and then purified by crystallization with methanol.

(*2R*,*3R*,*4S*,*5R*,*6R*)-2-(acetoxymethyl)-6-(4-((4-(€-3-(3,4-dimethoxyphenyl)acryloyl)-3-hydroxyphenoxy)methyl)-1H-1,2,3-triazol-1-yl)tetrahydro-2H-pyran-3,4,5-triyl triacetate (**7a**). Yellow solid; yield: 51%; m.p.: 169–171 °C (methanol); ^1^H NMR (CDCl_3_, 300.13 MHz), δ: 13.49 (1H, s, OH-2′), 7.89 (1H, s, H-3″), 7.86 (1H, d, J = 9.3 Hz, H-6′), 7.84 (1H, d, J = 15.5 Hz, H-β), 7.43 (1H, d, J = 15.5 Hz, H-α), 7.26–7.22 (1H, m, H-6), 7.16 (1H, d, J= 1.9 Hz, H-2), 6.90 (1H, d, J= 8.3 Hz, H-5), 6.58–6.55 (2H, m, H-3′ and H-5′), 5.90 (1H, d, J= 9.2 Hz, H-1‴), 5.48–5.38 (2H, m), 5.27–5.18 (1H, m) (H-2‴, H-3‴,H-4‴), 5.24 (2H, s, H-1″), 4.33–4.11 (2H, m, H-6‴), 4.04–3.98 (1H, m, H-5‴), 3.96 (3H, s, 3-OCH_3_), 3.93 (3H, s, 4-OCH_3_), 2.07, 2.06, 2.02, 1.86 (12H, s, 2‴, 3‴, 4‴, 6‴-COCH_3_). ^13^C NMR (CDCl_3_, 75.47 MHz) δ: 192.0 (CO), 170.6, 170.0, 169.5, 169.1 (2‴, 3‴, 4‴, 6‴-COCH_3_), 166.6 (C2′), 164.5 (C4′), 151.8 (C3), 149.4 (C4) 145.0 (Cβ), 144.1 (C2″), 131.4 (C6′), 127.8 (C1), 123.6 (C6), 121.5 (C3″), 118.0 (Cα), 114.7 (C1′), 111.3 (C5), 110.3 (C2), 107.9 (C5′), 102.3 (C3′), 85.9 (C1‴), 75.3 (C5‴), 72.7, 70.4, 67.8 (C2‴, C3‴, C4‴), 62.0 (C1″), 61.6 (C6‴), 56.2 (3-OCH_3_), 56.1 (4-OCH_3_), 20.8, 20.7, 20.6, 20.3 (2‴, 3‴, 4‴, 6‴-COCH_3_). HRMS (ESI^+^) *m*/*z* calcd for C_34_H_38_N_3_O_14_ [M + H^+^] 712.23483, found 712.23337.

(*2R*,*3R*,*4S*,*5R*,*6R*)-2-(acetoxymethyl)-6-(4-((3-hydroxy-4-((*E*)-3-(3,4,5-trimethoxyphenyl)acryloyl)phenoxy)methyl)-1H-1,2,3-triazol-1-yl)tetrahydro-2H-pyran-3,4,5-triyl triacetate (**7b**). Yellow solid; yield: 57%; m.p.: 127–128 °C (quamarnol); ^1^H NMR (CDCl_3_, 300.13 MHz), δ: 13.41 (1H, s, OH-2′), 7.89 (1H, s, H-3″), 7.87 (1H, d, J = 8.8 Hz, H-6′), 7.81 (1H, d, J = 15.4 Hz, H-β), 7.45 (1H, d, J = 15.4 Hz, H-α), 6.87 (2H, s, H-2 and H-6), 6.60–6.56 (2H, m, H-3′ and H-5′), 5.91–5.88 (1H, m, H-1‴), 5.48–5.39 (2H, m), 5.27–5.21 (1H, m) (H-2‴, H-3‴, H-4‴), 5.25 (2H, s, H-1″), 4.34–4.13 (2H, m, H-6‴), 4.03–4.00 (1H, m, H-5‴), 3.93 (6H, s, 3-OCH_3_ and 5-OCH_3_), 3.91 (3H, s, 4-OCH_3_), 2.08, 2.07, 2.02, 1.87 (12H, s, 2‴, 3‴, 4‴, 6‴-COCH_3_). ^13^C NMR (CDCl_3_, 75.47 MHz) δ: 191.9 (CO), 170.6, 170.0, 169.5, 169.1 (2‴, 3‴, 4‴, 6‴-COCH_3_), 166.6 (C2′), 164.6 (C4′), 153.7 (C3 and C5), 145.0 (Cβ), 144.1 (C2″), 140.8 (C4), 131.5 (C6′), 130.3 (C1), 121.5 (C3″), 119.5 (Cα), 114.7 (C1′), 108.0 (C5′), 105.9 (C2 and C6), 102.3 (C3′), 86.0 (C1‴), 75.4 (C5‴), 72.7, 70.4, 67.8 (C2‴, C3‴, C4‴), 62.1 (C1″), 61.6 (C6‴), 61.2 (4-OCH_3_), 56.4 (3-OCH_3_ and 5-OCH_3_), 20.8, 20.7, 20.6, 20.3 (2‴, 3‴, 4‴, 6‴-COCH_3_). HRMS (ESI^+^) *m*/*z* calcd for C_35_H_40_N_3_O_15_ [M + H^+^] 742.24539, found 742.24366.

#### 3.1.8. Synthesis of Chalcone-Triazolyl-Glycosides **8a** and **8b**

To a solution of **6a** (0.050 g, 0.15 mmol) or **6b** (0.2500 g, 0.68 mmol) and 2-azidoethyl-2,3,4,6-tetra-*O*-acetyl-β-D-glucopyranoside (0.123 g, 0.30 mmol or 0.566 g, 1.36 mmol) in THF/water solvent mixture (2:1; 30 mL), sodium ascorbate (0.117 g, 0.59 mmol or 0.538 g, 2.71 mmol) and copper(II) sulphate pentahydrate (0.074 g, 0.30 mmol or 0.339 g, 1.36 mmol) were added. The reaction mixture was heated for 1 h at 70 °C under MW irradiation of 250 W with agitation. After cooling, the reaction mixture was filtered and the THF in the filtrate was evaporated under reduced pressure. Then, the water suspension was extracted with ethyl acetate (2 × 20 mL) and the combined organic layers were washed with water (1 × 20 mL), dried over anhydrous sodium sulphate, concentrated under reduced pressure, and then purified by preparative TLC (SiO_2_; n-hexane: ethyl acetate, 2:8) (**8a**) or flash column chromatography (SiO_2_; n-hexane: ethyl acetate, 5:5) (**8b**).

(*2R*,*3R*,*4S*,*5R*,*6R*)-2-(acetoxymethyl)-6-(2-(4-((4-((E)-3-(3,4-dimethoxyphenyl)acryloyl)-3-hydroxyphenoxy)methyl)-1H-1,2,3-triazol-1-yl)ethoxy)tetrahydro-2H-pyran-3,4,5-triyl triacetate (**8a**). Yellow solid; yield: 65%; m.p.: 77–79 °C (n-hexane: ethyl acetate); ^1^H NMR (CDCl_3_, 300.13 MHz), δ: 13.49 (1H, s, OH-2′), 7.87 (1H, d, J = 9.7 Hz, H-6′), 7.85 (1H, d, J = 15.4 Hz, H-β), 7.73 (1H, s, H-3″), 7.44 (1H, d, J = 15.4 Hz, H-α), 7.25 (1H, dd, J = 8.1, 1.8, H-6), 7.16 (1H, d, J = 1.8, H-2), 6.91 (1H, d, J = 8.4, H-5), 6.60–6.56 (2H, m, H-3′ and H-5′), 5.23 (2H, s, H-1″), 5.18 (1H, t, J = 9.4), 5.06 (1H, t, J = 9.6), 5.03–4.97 (1H, m) (H-2⁗, H-3⁗, H-4⁗), 4.68–4.52 (2H, m, H-6⁗), 4.48 (1H, d, J = 7.9, H-1⁗), 4.27–4.21 (2H, m), 4.15–4.10 (1H, m), 3.90–3.81 (1H, m) (H-1‴, H-2‴), 3.96 (3H, s, 3-OCH_3_), 3.94 (3H, s, 4-OCH_3_), 3.72–3.66 (1H, m, H-5⁗), 2.08, 2.01, 1.99, 1.95 (12H, s, 2⁗, 3⁗, 4⁗, 6⁗-COCH_3_). ^13^C NMR (CDCl_3_, 75.47 MHz) δ: 192.0 (CO), 170.7, 170.3, 169.6, 169.5 (2⁗, 3⁗, 4⁗, 6⁗-COCH_3_), 166.6 (C2′), 164.6 (C4′), 151.8 (C4), 149.4 (C3) 144.9 (Cβ), 143.1 (C2″), 131.5 (C6′), 127.9 (C1), 124.6 (C3″), 123.6 (C6), 118.1 (Cα), 114.7 (C1′), 111.3 (C5), 110.3 (C2), 107.9 (C5′), 102.3 (C3′), 100.6 (C1⁗), 72.1 (C5⁗), 72.6, 71.1, 68.3 (C2⁗, C3⁗and C4⁗), 67.8, 61.8 (C1‴ and C2‴), 62.1 (C1″), 56.2 (3′-OCH_3_), 56.1 (4′-OCH_3_), 50.3 (C6⁗), 20.9, 20.7, 20.7, 20.7 (2⁗, 3⁗, 4⁗, 6⁗-COCH_3_). HRMS (ESI^+^) *m*/*z* calcd for C_36_H_42_N_3_O_15_ [M + H^+^] 756.261044, found 756.26373.

(*2R*,*3R*,*4S*,*5S*,*6S*)-2-(acetoxymethyl)-6-(2-(4-((3-hydroxy-4-((*E*)-3-(3,4,5-trimethoxyphenyl)acryloyl)phenoxy)methyl)-1H-1,2,3-triazol-1-yl)ethoxy)tetrahydro-2H-pyran-3,4,5-triyl triacetate (**8b**). Yellow solid; yield: 45%; m.p.: 78–81 °C (n-hexane: ethyl acetate); ^1^H NMR (CDCl_3_, 300.13 MHz), δ: 13.41 (1H, s, OH-2′), 7.87 (1H, d, J = 9.8 Hz, H-6′), 7.81 (1H, d, J = 15.4 Hz, H-β), 7.74 (1H, s, H-3″), 7.46 (1H, d, J = 15.4 Hz, H-α), 6.87 (2H, s, H-2 and H-6), 6.61–6.57 (2H, m, H-3′ and H-5′), 5.24 (2H, s, H-1″), 5.18 (1H, t, J= 9.4), 5.07 (1H, t, J = 9.6), 4.99 (1H, t, J = 8.7) (H-2⁗, H-3⁗, H-4⁗), 4.68–4.52 (2H, m, H-6⁗), 4.48 (1H, d, J = 7.9, H-1⁗), 4.28–4.21 (2H, m), 4.13–4.08 (2H, m) (H-1‴ and H-2‴), 3.93 (3H, s, 4-OCH_3_), 3.91 (6H, s, 3-OCH_3_ and 5-OCH_3_), 3.72–3.66 (1H, m, H-5⁗), 2.04, 2.02, 1.99, 1.95 (12H, s, 2⁗, 3⁗, 4⁗, 6⁗-COCH_3_). ^13^C NMR (CDCl_3_, 75.47 MHz) δ: 191.9 (CO), 170.7, 170.3, 169.6, 169.5 (2⁗, 3⁗, 4⁗, 6⁗-COCH_3_), 166.7 (C2′), 164.8 (C4′), 153.6 (C3 and C5), 144.9 (Cβ), 143.0 (C2″), 140.7 (C4), 131.5 (C6′), 130.4 (C1), 124.7 (C3″), 119.6 (Cα), 114.6 (C1′), 108.0 (C5′), 105.9 (C2 and C6), 102.3 (C3′), 100.6 (C1⁗), 72.1 (C5⁗), 72.6, 71.1, 68.3 (C2⁗, C3⁗ and C4⁗), 67.8, 61.9 (C1‴ and C2‴), 62.1 (C1″), 60.5 (4-OCH_3_), 56.4 (3-OCH_3_ and 5-OCH_3_), 50.3 (C6⁗), 21.2, 20.9, 20.7, 20.7 (2⁗, 3⁗, 4⁗, 6⁗-COCH_3_). HRMS (ESI^+^) *m*/*z* calcd for C_37_H_44_N_3_O_16_ [M + H^+^] 786.27161, found 786.26915.

#### 3.1.9. Synthesis of Acetophenone **9**

To a solution of 2,4-dihydroxyacetophenone (1.00 g, 6.57 mmol) and potassium carbonate (2.73 g, 19.72 mmol) in anhydrous acetone (20 mL), chloromethyl methyl ether (0.749 mL, 9.86 mmol) was added and the mixture refluxed for 1 h at 60 °C. Then, the reaction mixture was filtered, evaporated under reduced pressure and purified by flash column chromatography (SiO_2_; n-hexane: ethyl acetate, 9:1), giving rise to **9** with 84% yield. The structure elucidation of compound **9** was established by ^1^H and ^13^C NMR techniques and data were in accordance with previously reported results [55].

#### 3.1.10. Synthesis of Chalcones **10a** and **10b**

To a solution of **9** (0.500 g, 2.55 mmol) in methanol (20 mL) a solution of 40% NaOH in methanol was added until pH 14, under stirring. Then, a solution of 3,4-dimethoxybenzaldehyde (0.847 g, 5.10 mmol) or 3,4,5-trimethoxybenzaldehyde (1.00 g, 5.10 mmol) in methanol was slowly added to the reaction mixture. The reaction was submitted to MW irradiation at 180 W at 70 °C for 4 h. Then, a solution of 10% HCl was added until pH 5, and the obtained solid was filtered and washed with water and purified by crystallization with methanol, giving rise to **10a** and **10b** with 33% and 47% yield, respectively. The structure elucidation of both compounds was established by ^1^H and ^13^C NMR techniques and data of **10a** were in accordance with previously reported results [24]. Although the synthesis of compound **10b** has been previously reported [25], the NMR data are described here for the first tim€(*E*)-1-(2-hydroxy-4-(methoxymethoxy)phenyl)-3-(3,4,5-trimethoxyphenyl)prop-2-en-1-one (**10b**). Yellow solid; yield: 47%; m.p.: 132–134 °C (methanol); ^1^H NMR (CDCl_3_, 500 MHz), δ: 13.29 (1H, s, OH-2′), 7.85 (1H, d, J = 9.1 Hz, H-6′), 7.81 (1H, d, J = 15.4 Hz, H-β), 7.45 (1H, d, J = 15.4 Hz, H- α) 6.87 (2H, s, H-2 and H-6), 6.65 (1H, d, J = 2.4 Hz, H-3′), 6.60 (1H, dd, J = 9.0,2.5 Hz, H-5′), 5.23 (2H, s, H-1″), 3.93 (6H, s, 3-OCH_3_ and 5-OCH_3_), 3.91 (3H, s, 4-OCH_3_), 3.49 (3H, s, H-2″). ^13^C NMR (CDCl_3_, 120 MHz) δ: 192.0 (CO), 166.4 (C2′), 163.8 (C4′), 153.6 (C3 and C5), 144.9 (Cβ), 140.7 (C4), 131.4 (C6′), 130.4 (C1), 119.5 (Cα), 115.0 (C1′), 108.4 (C5′), 105.9 (C2 and C6), 104.1 (C3′), 94.2 (C1″), 61.2 (4-OCH_3_), 56.6 (C2″), 56.4 (3-OCH_3_ and 5-OCH_3_).

#### 3.1.11. Synthesis of Chalcones **11a** and **11b**

To a solution of **10a** (0.200 g, 0.58 mmol) or **10b** (0.250 g, 0.67 mmol) in methanol (10 mL), p-toluenesulfonic acid monohydrate (0.110 g, 0.58 mmol or 0.127 g, 0.67 mmol) was added. The reaction was submitted to conventional heating at 50 °C for 5 h. After the addiction of 10 mL of water, methanol was evaporated, and the aqueous solution was extracted with ethyl acetate (2 × 20 mL). The organic phase was washed with water (1 × 20 mL), dried over anhydrous sodium sulphate and concentrated under reduced pressure, giving rise to an orange solid. The crude product was purified by flash column chromatography (n-hexane: ethyl acetate, 8:2) (**11a**) or crystallization with chloroform (**11b**), giving rise to chalcone **11a** and **11b** with 24% and 31% yield, respectively. The structure elucidation of compounds **11a** and **11b** was established by ^1^H and ^13^C NMR techniques and data were in accordance with previously reported results [56,57].

#### 3.1.12. Synthesis of 2,3,4,6-Tetra-*O*-Acetyl-*β*-D-Glucopyranosyl Azide (**12**)

To a solution of 2,3,4,6-tetra-*O*-acetyl-α-D-glucopyranosyl bromide (1.00 g, 2.43 mmol) in acetone: water (9 mL, 2:1), sodium azide (0.197 g, 3.04 mmol) was added and the reaction mixture stirred at room temperature for 3 h. After, the acetone was evaporated under reduced pressure and a white solid was filtered. Crystallization of the solid with ethanol afforded 2,3,4,6-tetra-O-acetyl-*α*-D-glucopyranosyl azide **12** with 72% yield. The structure elucidation was established by ^1^H and ^13^C NMR techniques and data were in accordance with previously reported results [27].

### 3.2. Mussel (Mytilus galloprovincialis) Larvae Anti-Settlement Activity

Mussel (*Mytilus galloprovincialis*) plantigrades were collected in juvenile aggregates during low neap tides at Memória beach, Matosinhos, Portugal (41°13′59″ N; 8°43′28″ W). In laboratory, mussel plantigrade larvae (0.5–2 mm) were isolated in a binocular magnifier (Olympus SZX2-ILLT, Tokyo, Japan) to a petri dish with filtered seawater, and those with functional foot and competent exploring behaviour were selected for the bioassays. The flavonoids were screened at 50 µM in 24-well microplates with 4 well replicates per condition and 5 larvae per well, for 15 h, in the darkness at 18 ± 1 °C, following Almeida et al. (2015) [58]. Test solutions were obtained by dilution of the compounds stock solutions (50 mM) in DMSO and prepared with filtered seawater. All bioassays included a negative control with DMSO and a positive control with CuSO_4_, a potent AF agent. After the exposure period, the anti-settlement activity was determined by the presence/absence of attached byssal threads produced by each individual larvae.

All compounds that caused more than 60% of settlement inhibition (≤40% of settlement) in the screening bioassay were considered active and selected for the determination of the semi-maximum response concentration that inhibited 50% of larval settlement (EC_50_), at compounds concentrations of 3.12, 6.25, 12.5, 25, 50, 100, 200 μM.

### 3.3. Quantitative Structure–Activity Relationship

The eighteen flavonoid derivatives (**1a**, **1b**, **2a**, **2b**, **3a**, **3b**, **4a**, **4b** from Scheme 1; **6a**, **6b**, **7a**, **7b**, **8a**, **8b** from Scheme 2; and **10a**, **10b**, **11a**, **11b** from Scheme 3) were used to build a QSAR model using the experimental data obtained from the mussel (*Mytilus galloprovincialis*) larvae anti-settlement activity in vivo bioassay (AF activity = log(100/%settlement). AF activity was selected as a dependent variable in the QSAR analysis. The 18 molecules were randomly distributed into a training set (15 molecules) and a test set (3 molecules). CODESSA software (version 2.7.10, University of Florida, Gainesville, FL, USA) was used to calculate more than 500 constitutional, topological, geometrical, electrostatic, quantum-chemical and thermodynamical molecular descriptors [59]. The heuristic multilinear regression methodology was chosen to perform a complete search for the best multilinear correlations with a multitude of descriptors of the training set [60]. The 2D-QSAR model with the best square of the correlation coefficient (R^2^), F-test (F), and squared standard error (S^2^) was selected. The final model was further validated using the test set and leave-one-out (LOO) internal validation.

### 3.4. Inhibitory Activity against Biofilm-Forming Marine Bacteria Growth

For anti-bacterial screening, five strains of marine biofilm-forming bacteria from the Spanish Type Culture Collection (CECT): *Cobetia marina* CECT 4278, *Vibrio harveyi* CECT 525, *Halomonas aquamarina* CECT 5000, *Pseudoalteromonas atlantica* CECT 570, and *Roseobacter litoralis* CECT 5395 were used. Bacteria were inoculated and incubated for 24 h at 26 °C in marine broth (Difco) at an initial density of 0.1 (OD600) in 96 well flat-bottom microtiter plates and exposed to the test compounds at 15 μM. Test solutions were obtained by dilution of the compounds stock solutions (50 mM) in DMSO. Bacterial growth inhibition in the presence of the compounds was determined in quadruplicate at 600 nm using a microplate reader (Biotek Synergy HT, Vermont, USA). Negative and positive controls used were a solution of marine broth with DMSO, and a solution of marine broth with penicillin–streptomycin–neomycin, respectively. Compounds exerting a significant anti-bacterial activity (Dunnet test, *p* < 0.05) in the screening bioassays were selected for the determination of the effective inhibitory concentration (EC_50_).

### 3.5. Inhibitory Activity against Biofilm-Forming Marine Diatom Growth

The anti-microalgal activity of the most promising compounds was also evaluated against a benthic marine diatom, *Navicula* sp., purchased from the (Telde, Gran Canaria) Spanish Collection of Algae (BEA). Diatom cells were inoculated in f/2 medium (Sigma) at an initial concentration of 2–4 × 106 cells mL^−1^ and grown in 96-well flat-bottom microtiter plates for 10 days at 20 °C. *Navicula* growth inhibition in the presence of each compound at 15 μM was determined in quadruplicate and quantified based on the difference in cell densities among the treatments, and cells were counted using a Neubauer counting chamber. A positive control with cycloheximide (3.55 μM) and a negative control with f/2 medium 0.1% DMSO were included. Compounds that showed significant inhibitory activity in the screening assay (Dunnet test, *p* < 0.05) were selected for further determination of their effective inhibitory concentrations (EC_50_).

### 3.6. In Vitro Acetylcholinesterase (AChE) and Tyrosinase (Tyr) Activities

The ability of the most promising compounds to inhibit AChE and Tyr was tested to assess their potential mode of action related with neurotransmission disruption or impairment of adhesive metabolism pathways, respectively.

AChE activity was evaluated using Electrophorus electric AChE Type V-S (SIGMA C2888, E.C. 3.1.1.7), according to Ellman et al. (1961) [61] with some modifications [58,62]. Reaction solution containing 1 M phosphate buffer pH 7.2, 10 mM dithiobisnitrobenzoate (DTNB) (acid dithiobisnitrobenzoate and sodium hydrogen carbonate in phosphate buffer) and 0.075 M acetylcholine iodide was added to pure AChE enzyme (0.25 U/mL) and each test compound (final concentration of 25, 50 and 100 μM, 1% DMSO) in quadruplicate. All tests included a positive control with eserine (200 μM, water) and a negative control with 1% DMSO in water. The optical density was measured at 412 nm in a microplate reader (Biotek Synergy HT, Winooski, Vermont, USA) during 5 min at 25 °C.

Tyr inhibition assay was performed using *Agaricus bisporus* Tyr (EC1.14.18.1) according to Adhikari et al. (2008) [63] with some modifications [8]. The enzymatic reaction follows the catalytic conversion of L-Dopa to dopaquinone and the formation of dopachrome by measuring the absorbance at 475 nm. Briefly, Tyr (25 U/mL) was added to 50 mM phosphate buffer pH 6.5 and the tested compounds at 25, 50 and 100 μM (final concentrations, 1% DMSO). The enzymatic activity was triggered by the addition of L-dopa (25 mM). Kojic acid (1.4 mM, water) was included as positive control and 1% DMSO in water as negative control.

### 3.7. Environmental Fate Parameters: Artemia Salina Ecotoxicity Bioassay

The brine shrimp (*Artemia salina*) nauplii lethality test was used to determine the toxicity of promising AF compounds to non-target organisms [64]. *Artemia salina* eggs were allowed to hatch in seawater for 48 h at 25 °C. Bioassays were performed in 96-wells microplates with 15–20 nauplii per well and 200 μL of the compounds test solution. Compounds were tested at final concentrations of 25 and 50 μM (filtered seawater with 1% DMSO). All tests included K_2_Cr_2_O_7_ (13.6 μM) as positive control and DMSO (1%) as negative control. Bioassays were run in the dark at 25 °C, and the percentage of mortality was determined after 48 h of exposure.

### 3.8. Statistical Analysis

Datasets from anti-settlement, antibacterial and anti-microalgal bioassay, and determination of AChE and Tyr activities, were analysed by one-way analysis of variance (ANOVA) followed by a multi-comparisons Dunnett’s test against negative control (*p* < 0.05). For the AF bioassays, the half maximum response concentration (EC_50_) values for each compound, when applicable, were calculated using Probit regression analysis. Significance was considered at *p* < 0.01, and 95% lower and upper confidence limits (95%LCL; UCL). The software IBM SPSS Statistics 26 (Armonk, New York, USA) was used for statistical analysis.

## 4. Conclusions

In this study, eight new triazole-flavonoid hybrids were synthesized using the click chemistry approach. From the series of synthesized compounds, flavone **1b** and chalcones **7b**, **11a** and **11b** showed significant anti-settlement activity towards the macrofouling species *Mytilus galloprovincialis* adhesive larvae. Regarding the compounds’ structures, HACA1 was the most significant descriptor for the obtained QSAR model, contributing positively to the AF activity. Particularly, triazolyl glycosylated chalcone **7b**, with a high number of hydrogen-bonding acceptors, showed the most effective anti-settlement activity (EC_50_ = 3.28 µM; 2.43 µg·mL^−1^) with the highest therapeutic ratio (LC_50_/EC_50_ > 60.98), exhibiting also a significant inhibitory activity against the marine diatom *Navicula* sp. (EC_50_ = 41.76 µM; 30.94 µg·mL^−1^), suggesting potential in the suppression of biofouling colonization succession. Flavone **1b**, which was effective against the settlement of mussel larvae, also showed capacity to inhibit the activity of Tyr, which might explain the specific AF activity against mussel larvae. Ecotoxicity studies on the non-target species *Artemia salina* revealed that the flavonoids **1b**, **7b**, **11a** and **11b** did not show ecotoxicity to the nauplii of this sensitive crustacean, even at 50 µM, a concentration much higher than their EC_50_. These results disclosed synthetic flavonoids, particularly a new chalcone incorporating a 1,2,3- triazole ring (**7b**), with potential to be a good environmentally compatible alternative to the majority of the antifoulants in use. Flavonoids are ubiquitous in Nature and, therefore, they come with the advantage that they have been selected during evolution to have high specificity, high efficiency and some might be potential nontoxic inhibitors of fouling. Natural compounds are usually biodegradable, not leaving residue in the environment, and are thus considered one of the most promising alternatives to the biocides in use. However, the yields of natural compounds from marine organisms are generally poor, hindering their development as AF agents. Moreover, optimizing a micro-organism for enhanced production of antifoulant is generally laborious and time consuming. Synthesis of nature-like antifoulants seems to be a more sustainable way to create an opportunity to produce commercial supplies for the antifouling industry.

## Figures and Tables

**Table 1:** Antifouling (AF) effectiveness and toxicity parameters of flavones **1b**, **4a** and **4b** and chalcones **6a**, **6b**, **7b**, **8a**, **8b**, **11a** and **11b** towards mussel plantigrade larvae.

Compound	EC_50_ (μM)	EC_50_ (μg·mL^−1^)	LC_50_ (µM)	LC_50_/EC_50_
**1b**	8.34 (95% CI: 4.2–13.36)	2.87	> 200	> 23.98
**4a**	42.55 (95% CI: 34.90–52.80)	32.75	> 200	> 4.70
**4b**	48.22 (95% CI: 30.57–85.40)	38.56	> 200	> 4.15
**6a**	84.52 (95% CI: 45.07–267.02)	28.60	> 200	> 2.37
**6b**	85.56 (95% CI: 44.84–291.41)	31.52	> 200	> 2.34
**7b**	3.28 (95% CI: 1.97–4.74)	2.43	> 200	> 60.98
**8a**	35.83 (95% CI: 19.22–74.74)	27.07	> 200	> 5.58
**8b**	53.90 (95% CI: 29.98–126.88)	42.35	> 200	> 3.71
**11a**	18.10 (95% CI: 13.95–23.44)	5.44	> 200	> 11.05
**11b**	9.64 (95% CI: 3.85–17.22)	3.18	> 200	> 20.75

EC_50_: minimum concentration that inhibited 50% of larval settlement; LC_50:_ median lethal dose; LC_50_/EC_50:_ therapeutic ratio_;_ CI: confidence interval. EC_50_ are recommend to be less than 25 µg/mL and therapeutic ratio higher than 15 for effective AF agents [31].

## Data Availability

Data is contained within the article
